# Protocol to study how expectations guide predictive eye movements and information sampling in humans

**DOI:** 10.1016/j.xpro.2025.103737

**Published:** 2025-04-08

**Authors:** Annika Garlichs, Mark Lustig, Matthias Gamer, Helen Blank

**Affiliations:** 1 Department of Systems Neuroscience, University Medical Center Hamburg-Eppendorf, Hamburg, Germany; 2 Hamburg Brain School, University Medical Center Hamburg-Eppendorf, Hamburg, Germany; 3 Department of Psychology, University of Hamburg, Hamburg, Germany; 4 Department of Psychology, University of Würzburg, Würzburg, Germany; 5 Research Center One Health Ruhr of the University Alliance Ruhr, Faculty of Psychology, Ruhr-University Bochum, Bochum, Germany; 6 Predictive Cognition, Faculty of Psychology, Ruhr-University Bochum, Bochum, Germany

**Keywords:** Neuroscience, Cognitive neuroscience, Behavior

## Abstract

Investigating eye movements provides a unique tool to explore how expectations influence information sampling in the visual domain. Here, we present a protocol for measuring predictive eye movements during face anticipation as well as fixations and dwell time during face recognition in humans. We describe steps for setting up two eye-tracking experiments. We then detail procedures for the measurement and analysis of eye-tracking data.

For complete details on the use and execution of this protocol, please refer to Garlichs et al.^1^

## Highlights

• Guide for studying expectation effects on information sampling with eye tracking
• Steps for setting up, running, and analyzing predictive eye-tracking experiments
• Instructions on measuring eye movements in anticipation and recognition tasks

## Abstract (editor highlights)

Publisher’s note: Undertaking any experimental protocol requires adherence to local institutional guidelines for laboratory safety and ethics.

## Graphical abstract

**Figure:**
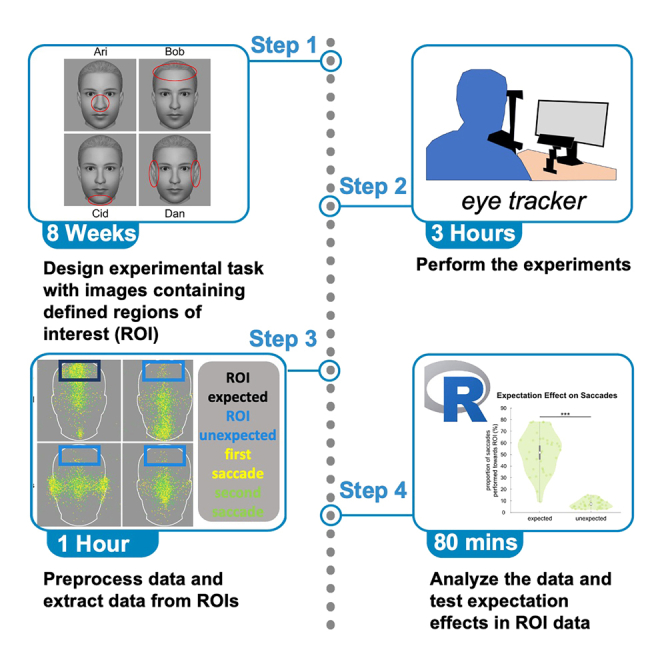

## Before you begin

### Institutional permissions

This study protocol was approved by the Ethics Committee of the Chamber of Physicians in Hamburg. Any subsequent studies will need permission from the relevant institutions.

### Investigating eye movements to test expectation effects on information sampling—Description of analysis strategy

**Figure 1:**
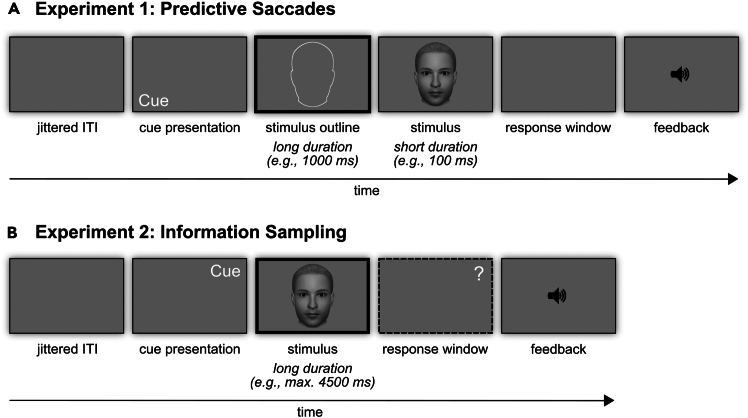
Experimental design (A) Exemplary trial of Experiment 1: After the presentation of a prior cue, an outline of the stimulus is presented. Next, a stimulus is only briefly shown (e.g., for 100 ms). Engage participants in a task to assure usage of the cue. Provide feedback, e.g., via a tone. The thick black frame indicates the time window for the eye-tracking analyses. ITI, inter-trial interval (e.g., jittered). (B) Exemplary trial of Experiment 2: in contrast to Experiment 1, the stimulus is presented longer (e.g., up to 4,500 ms or until a button press). Engage participants in a task to use the predictive information. Provide feedback, e.g., via a tone. The thick black frame indicates the time window for the eye-tracking analyses.

Analyzing eye movements offers a valuable method for studying how expectations shape information sampling.^2^ This protocol aims to describe this generic effect in the specific domain of face processing as faces represent the most important visual social stimulus and face processing has been shown to be fundamentally influenced by contextual factors.^1^^,^^3^^,^^4^ To induce expectations, participants can be trained to associate cues with an upcoming image with specific features. Eye-tracking data are collected to examine how these expectations influence eye movements during both the anticipation and perception of images in two separate experimental setups. The protocol is applicable to a broad range of experiments where participants learn associations between cues and specific spatial regions. In the current protocol, we specifically describe experiments using images of faces and refer to our recent study^1^ as an example in which each face is characterized by a distinct feature like a high forehead or wide chin. In each trial, expectations about the face and its distinct feature are induced by a preceding name cue. In the test phase, the approach uses face morphs between two identities, always containing the cued expected and one unexpected identity (see Figure 1). Experiment 1 examines whether participants use context information to make predictive saccades toward expected facial features by introducing an anticipation phase before the face image appears. Experiment 2 explores whether expectations affect how participants sample face information. This approach allows testing whether expectations guide active sampling, where participants fixate on expected facial features first and spend more time on those features in face morphs containing expectation-congruent and -incongruent information. The protocol involves the following 4 major parts, each of them consisting of several steps:

i. Design experimental task with images containing defined regions of interest (ROIs)
ii. Perform the experiment
iii. Preprocess the data
iv. Analyze the data

### Design experimental task

**Timing: 8 weeks**

The provided timing estimates are based on our experience with the experiments reported in Garlichs et al.^1^ Please note that actual time requirements may vary from our suggestions of approximately 8 weeks for designing and programming the experiment and 90 min for collecting data of one participant depending on the specific choice of stimuli and experimental setup.

1. Choose an experimental task to answer your research questions.

***Note:*** There are several things to consider when designing a task for eye-tracking experiments. Some of the points below apply to eye-tracking analyses in general; others are specific to this protocol.

**CRITICAL:** Specifically, the duration of intervals before, during, and after the visually presented stimuli is crucial and has to be chosen according to your research question to allow or prevent eye movements during each phase. Take into account that a saccade typically takes up to 20–40 ms^5^ and a fixation typically ranges from 150 to 300 ms.^6^ For example, in our specific study, each trial began with a jittered inter-trial interval between 1.25 and 1.75 s (mean: 1.50 s) followed by a name cue presented for 750 ms (see Figure 1). Afterwards, either a face outline was shown for 1,000 ms (inter-stimulus interval, ISI) to measure anticipatory eye movements before a brief presentation of a face (100 ms) followed (Experiment 1) or a face was shown for 4,500 ms (or until a participant’s response) to measure eye movements during face recognition (Experiment 2). Next, a response window of 1,500 ms and an auditory feedback window of 750 ms were presented.

**CRITICAL:** To induce expectations experimentally, make sure that participants learn the cue-stimulus associations (e.g., name-face) beforehand. In our pilots and main experiments, we had success with having participants learn to associate the four distinct faces with their respective names in a preceding training session lasting about 15 min.

***Note:*** Allow sufficient breaks so that participants can relax their eyes, e.g., by dividing the training session into several blocks of about 5 min. Build up the information during the training so that it can be learned in succession. Our training was divided into three blocks. In the first block, provide feedback by calling attention to a given distinct feature (e.g., the wide chin of a face) by highlighting it with a red circle, showing written text at the bottom of the screen, and playing a tone, which was identical for all faces, indicating whether the response was correct, incorrect, or too slow. In the second block, you can shorten the duration of the face presentation. In the third block, only provide auditory feedback. After the final block, provide verbal feedback about the accuracy and, if it is below 75%, repeat the last training block up to two times. If the threshold can still not be surpassed, do not let the participant proceed to the main experiment.

***Note:*** Conduct a shortened version of the main experiment to familiarize participants with the trial structure and the corresponding cue-stimulus contingencies. In our study, we used short versions of about 2 min: in 1/3 of the trials, the name was followed by the cued expected identity, in 1/3 of the trials by an unexpected identity, and in 1/3 of the trials by a face morph between the expected and an unexpected identity. Participants were familiarized with the task of the main experiment to classify the presented face as the ‘expected’ or an ‘unexpected’ identity and, in Experiment 2, to identify an ‘unexpected’ face as one of the four learned identities.

**CRITICAL:** The choice of visual stimuli is important for an eye-tracking experiment. Choose the luminance and size of your images carefully. We used grayscale images of four male faces created with FaceMaker (http://facemaker.uvrg.org/).^7^ Also ensure that stimuli are large enough to induce eye movements. In our experiment, the size of the images was 1,100 × 1,100 pixels, with the face covering approximately 736 × 1,064 pixels (width x height) of the screen with 1,600 × 1,200 px and a display size of 41.0 × 31.0 cm. Distance from the eyes to the screen was ∼560 mm, leading to a visual angle of 19.17 × 27.44° for the face stimuli.

2. Define regions of interest (ROIs).

***Note:*** Make sure that the size of the ROIs is equal or account for differences in size during analyses because the probability of a saccade randomly landing in an ROI depends on its area. As an example, our ROIs were four rectangular regions with the same area, covering the distinct features of the four identities (i.e., forehead, chin, ears, and nose). ROIs should also be matched for eccentricity as much as possible to avoid the central viewing tendency issue, as discussed later under ‘Limitations’.

3. Estimate the required sample size to reveal a significant effect in your respective analysis.

***Note:*** In our experiment, sample sizes for both experiments (*N* = 34, each) were preregistered and based on power analyses. For Experiment 1, the sample size was optimized to obtain .80 power to detect a medium effect size (*f* = 0.25) at .05 alpha error probability for testing whether participants perform more predictive saccades to an ROI if its facial feature has been expected compared to when it has not been expected, as the main effect ‘expectation’ in a 2 ✕ 2 repeated measures ANOVA with the within-subject factors ‘expectation’ (expected, unexpected) and ‘saccade’ (first, second). For Experiment 2, we aimed to optimize the power for our main research questions regarding the face morphs: we tested whether the dwell time on the expected ROI was significantly different from the dwell time on (1) the ROI associated with the other identity contained in a face morph, and on (2) the other ROIs using paired *t*-tests (two-sided) with a power of .80 to detect a medium effect size (dz = 0.50) at .05 alpha error probability.

***Note:*** Such power analysis can be carried out with G∗Power.^8^

## Key resources table

**Table:** 

REAGENT or RESOURCE	SOURCE	IDENTIFIER
**Other**		

EyeLink 1000 eye-tracking device	SR Research	https://www.sr-research.com/

**Deposited data**		

Experimental data (Experiment 1)	own	https://doi.org/10.17605/OSF.IO/7E38V
Stimuli (Experiment 1)	own	https://doi.org/10.17605/OSF.IO/7E38V
Material for experiments (Experiment 1)	own	https://doi.org/10.17605/OSF.IO/7E38V
Code for analyses (Experiment 1)	own	https://doi.org/10.17605/OSF.IO/7E38V
Code for experiments (Experiment 1)	own	https://doi.org/10.17605/OSF.IO/7E38V
Experimental data (Experiment 2)	own	https://doi.org/10.17605/OSF.IO/TBDH6
Stimuli (Experiment 2)	own	https://doi.org/10.17605/OSF.IO/TBDH6
Material for experiments (Experiment 2)	own	https://doi.org/10.17605/OSF.IO/TBDH6
Code for analyses (Experiment 2)	own	https://doi.org/10.17605/OSF.IO/TBDH6
Code for experiments (Experiment 2)	own	https://doi.org/10.17605/OSF.IO/TBDH6

**Software and algorithms**		

R (v.4.2.0)	R Core Team	https://www.r-project.org
RStudio (v.2022.02.2)	RStudio Team	https://posit.co
MATLAB (v.R2020b)	MathWorks	https://de.mathworks.com
faceMaker	Schwind et al.^7^	http://facemaker.uvrg.org/
Audacity (v.3.0.0)	Audacity Team	http://audacity.sourceforge.net/

## Materials and equipment

In our experiments, eye movements are recorded from the participant’s right eye using an EyeLink 1000 system at a sampling rate of 1,000 Hz. Please adjust your procedure if you use other equipment, record both eyes, or use another sampling rate. We recommend adhering to general guidelines^9^^,^^10^ and fixing the participant’s head position using a chin rest and forehead bar.

## Step-by-step method details

### Perform the experiment

**Timing: 90 min (Experiment 1) + 90 min (Experiment 2)**

1. Set up the experimental room and position the participant in front of the eye tracker.
  a. Start stimulus presentation PC and eye-tracking recording PC.
    ***Note:*** Turn on the eye-tracking equipment at least 5 min before use, and good practice is to turn on all stimulus displays at least 30 min before use. This should be done before the participant is seated at the eye tracker.
  b. Let the participant sign the consent forms and fill in any demographic data forms (e.g., age, self-reported gender).
  c. Make sure that the participant has normal or corrected-to-normal vision.
    ***Note:*** Soft contact lenses usually work fine, glasses may require some adjustments to the eye tracker to deal with reflections. If applicable, instruct the subject to remove makeup (e.g., mascara) from the eye region.
  d. Instruct the participant to have a seat at the eye tracker, so that their chin and forehead are in contact with the chin rest and the forehead bar.
    ***Note:*** Ask them to adjust the seat height until they feel comfortable.
  e. Explain the marked keys on the keyboard and hand them the headphones.
  f. Control your light settings to be constant and comparable for all participants, e.g., close the window shade and turn on the desk lamp.
  g. Leave the room (if possible, i.e., if you have a separate control room in which the stimulus PC is located).
2. Train participants to learn the associations between any types of cues, such as written cues or auditory tones, and the subsequently presented visual stimuli.

***Note:*** In our specific experiment, we used written name cues and faces.

3. After the training is finished, evaluate the performance of the last block.

***Note:*** The task was to identify the presented face as one of the four learned faces by pressing one of the four corresponding buttons.

**CRITICAL:** If performance is above your pre-specified criterion (e.g., we used 75% correct responses), you can continue with the next step. If performance is below your pre-specified criterion, you should repeat the last block of the training up to two times.

***Optional:*** This is a good time to check any input devices the participants will use throughout the task and run a shortened version of the main experiment to familiarize participants with the setup.

***Optional:*** You could include a short repetition of the cue-stimulus association training between blocks of the main experiment to ensure clear mental representations of the stimuli (e.g., of the faces). In our study, in Experiment 2, we conducted a short repetition after the second experimental block (out of four blocks).

4. Calibrate the eye tracker.
  a. Visually check if pupil and corneal reflection are correctly identified by the tracker: they should be marked in dark and light blue, respectively (when you are using the EyeLink setup).
    ***Note:*** If that is not the case, use the Auto Threshold button in the upper left corner or the arrow buttons underneath to adjust the thresholds (when you are using the EyeLink setup).
    ***Note:*** By far the most common issue is dark eyelashes being identified as pupil area. This can lead to a variety of issues, so make sure this is not the case (if possible).
  b. Instruct the participant.
    ***Note:*** We used verbal instructions: ‘We will calibrate the eye tracker in a moment. Points will appear on the screen. Please look at them until they disappear. But first, I will adjust the focus. Please press the spacebar.’
  c. Perform a calibration and a subsequent validation using a sufficient number of calibration points at the start of each experimental block.
    ***Note:*** In our study, we used a 13-point-grid.
    **CRITICAL:** Repeat the validation step until the result was at least ‘good’ according to the guidelines of the manufacturer (i.e., worst point error < 1.5°, average error < 1.0°). In case of calibration issues, see troubleshooting, problem 1.
  d. Move quickly from calibration to the experiment, so any task instructions should ideally be provided before step #3.
    ***Note:*** Participants should be encouraged to keep their heads still after the calibration.
    ***Note:*** Now follows the major part and the participant performs the experimental task and all data needed for the analysis are gathered. The participant should receive all necessary instructions to perform the task on-screen.
5. Run the main eye-tracking experiment.
  a. Repeat the calibration and validation step between blocks.

### Preprocess data

**Timing: 30 min (Experiment 1) + 30 min (Experiment 2)**

***Note:*** There are many protocols for preprocessing eye-tracking data in various software packages (e.g., R, or from SR Research). The protocol below represents a fairly standard way of preprocessing eye-tracking data^9^ and you can find the preprocessing code used in this protocol in the code repository.

***Note:*** Our script uses the R package 'eyelinkReader'. This package requires that you have the EyeLink Developers Kit installed on your device. Therefore, before being able to run that script, you need to download the EyeLink Developers Kit from www.sr-support.com. Further instructions are supplied by the developer of the eyelinkReader package (https://github.com/alexander-pastukhov/eyelinkReader). If you have installed the above-mentioned packages, you can read in the raw EyeLink data file (.edf) into R using the ‘read_edf()’ function. An eyelinkRecording object will be created in your R environment containing detailed information about saccades, fixations, and blinks.

6. Extract saccades during the ISI, i.e., the anticipation phase with the stimulus outline, for analyzing anticipatory eye movements.
  ***Note:*** A saccade should be considered valid when all of the following criteria are met:
  a. The end coordinates of the saccade (genx, geny) are located in the stimulus region (i.e., the face ROI in the current experiment).
  b. The saccade starts after ISI onset + 100 ms (delay introduced for intentional saccades).
  c. The apparent saccade is not wrongly detected because of a blink.
    ***Note:*** Blinks are automatically detected by the EyeLink 1000 during recording and are read-out by the eyelinkReader-package. Blinks are events in which the pupil size is very small, the pupil is missing in the camera image or is severely distorted by eyelid occlusion. Blinks are often preceded and followed by partial pupil occlusion that causes the tracker to (erroneously) register changes in pupil position. Depending on the eye-tracking system that is used, these are often detected as saccades surrounding blinks which do not represent real changes in gaze position and should therefore be discarded.
    ***Note:*** Saccades were defined as periods in which the velocity exceeded 30°/s or the acceleration 8,000°/s^2^, respectively. The saccadic motion threshold was set to 0.1°, which is within the range suggested by the manufacturer (0.1°–0.2°). The velocity and acceleration thresholds are recommendations of the manufacturer for reading and cognitive research, such as our face study. For experiments investigating smooth pursuit and psychophysical research, more liberal thresholds such as 22°/s and 4,000°/s^2^ are recommended.
    ***Note:*** Fixations were automatically detected by the EyeLink 1000 system as periods of stable gaze between saccades based on the above-mentioned velocity, acceleration, and dispersion thresholds. For systems with lower sampling rate, different algorithms for parsing the eye-tracking data might be appropriate.^9^
7. Extract fixations during face presentation (average coordinates: gavx, gavy).
  ***Note:*** A fixation should be considered valid when one of the following criteria is met:
  a. The fixation occurs entirely within the face presentation window.
  b. The fixation smears into the face presentation window (in this case, cut the fixation onset to face start).
  c. The fixation smears out of the face presentation window (in this case, cut the fixation offset to the end).
  d. The fixation smears into and out of the face presentation window (in this case, cut the fixation onset to face start and the fixation offset to face end).
8. Evaluate the exclusion criteria: Check your pre-specified exclusion criteria.
  ***Note:*** We used the following steps and criteria:
  a. Single-trial exclusion if the name cue was not fixated (a prerequisite for intentional saccades during the ISI, i.e., the anticipation phase with the stimulus outline).
  b. Single-trial exclusion if no response was given.
  c. Single-trial exclusion if >30% invalid data (e.g., due to blinks or pupil loss) during the ISI, i.e., the anticipation phase with the 'base face' outline for 1,000 ms (Experiment 1).
  d. Single-trial exclusion if >50% invalid data during face presentation (Experiment 2).
  e. Whole data set exclusion if >30% invalid trials.

### Analyze data

**Timing: 15 min (Experiment 1) + 20 min (Experiment 2) + 45 min for supplementary analyses for Experiment 2**

9. Perform the main analysis on predictive saccades, defined as the saccades that occur during the anticipatory phase of the task (Experiment 1).
  a. Count how many predictive saccades landed in an ROI if the corresponding facial feature was expected versus when it was unexpected during anticipation of the face image.

***Note:*** Here, we provide illustrative code with ROI#1 in this example as a simplified snippet of actual code used in ‘Experiment_1_predictive_saccades.R’ (lines 167-186) as found in the OSF repository. Note that the dimensions of the ROI are given as placeholders that need to be defined elsewhere.

> # loop over participants, blocks, and trials

> # loop through saccades

> for (i_row in (1:nrow(ISI_saccades_first_second))) {

> # get information which face was expected

> priorID <- ISI_saccades_first_second$prior_ID[i_row]

>

> # e.g., ROI#1 as example

> if ((ISI_saccades_first_second$genx[i_row]>= ROI1dim_x_min) &

> (ISI_saccades_first_second$genx[i_row]<= ROI1dim_x_max) &

> (ISI_saccades_first_second$geny[i_row]>= ROI1dim_y_min) &

> (ISI_saccades_first_second$geny[i_row]<= ROI1dim_y_max)) {

> ISI_saccades_first_second$ROI[i_row] <- 1

> # if it was the expected ROI

> if (priorID==1) {

> ISI_saccades_first_second$expected[i_row] <- 1

> } else {

> ISI_saccades_first_second$expected[i_row] <- 0

> }

> }

10. Perform the main analysis on fixation order during the presentation of face images, 1) in all trials, 2) in face morph trials, 3) in *mismatch* trials in which an unexpected face was shown.

***Note:*** Here, we provide illustrative code, as a simplified snippet of actual code used in ‘Experiment_2_order_effect.R’ (lines 462-492) as found in the OSF repository.

> # loop over participants, blocks, and trials

> # check if there was a fixation for that trial

> if (nrow(allFix_perROI_perTrial)>0) {

> ROI_numbers <- c(1:4)

> # extract when each ROI was fixated first

> firstFix_inROI_perTrial<- distinct(allFix_perROI_perTrial, > ROI, .keep_all = TRUE)

> # order the remaining fixations

> nrow_order_count <- 1

> for (i_row in (1:nrow(firstFix_inROI_perTrial))) {

> firstFix_inROI_perTrial$order[i_row] <- nrow_order_count

> nrow_order_count <- nrow_order_count+1

> }

> # add the fixation information into a new data frame

> if (nrow(firstFix_inROI_perTrial)==1) {

> firstFix_inROI_perTrial_order[row_count,] <-

> firstFix_inROI_perTrial[1,]

> } else if (nrow(firstFix_inROI_perTrial)==2) {

> firstFix_perROI_perTrial_order[row_count:(row_count+1),] <-

> firstFix_inROI_perTrial[1:2,]

> } else if (nrow(firstFix_inROI_perTrial)==3) {

> firstFix_inROI_perTrial_order[row_count:(row_count+2),] <-

> firstFix_inROI_perTrial[1:3,]

> } else if (nrow(firstFix_inROI_perTrial)==4) {

> firstFix_perROI_perTrial_order[row_count:(row_count+3),] <-

> firstFix_inROI_perTrial[1:4,]

 }

> # add randomly assigned order for missing ROIs

> # extract existing ROIs

> fixated_ROIs <- firstFix_inROI_perTrial$ROI

> # which ROIs are still missing?

> remaining_ROIs <- setdiff(ROI_numbers, fixated_ROIs)

> }

11. Perform the main analysis on fixations during the presentation of morphed face images (Experiment 2).
  a. Count how many (and quantify how long) fixations landed in the expected, unexpected, and other two ROIs (i.e., regions that are not expected and do not contain unexpected features) during the presentation of the face image.

***Note:*** Here, we provide illustrative code, as simplified snippets of actual code used in ‘Experiment_2_nfix_and_dwell_time.R’ (lines 118-148 and 643-664) as found in the OSF repository.

> # number of fixations

> # loop over participants, blocks, and trials

> # loop through fixations

> for (i_row in (1:nrow(face_fixations_morphs))) {

> # get information which face was expected

> priorID <- face_fixations_morphs$prior_ID[i_row]

>

> # e.g. ROI#1

> if ((face_fixations_morphs$ gavx[i_row]>= ROI1dim_x_min) &

> (face_fixations_morphs$ gavx[i_row]<= ROI1dim_x_max) &

> (face_fixations_morphs$ gavx[i_row]>= ROI1dim_y_min) &

> (face_fixations_morphs$ gavx[i_row]<= ROI1dim_y_max)) {

> face_fixations_morphs $ROI[i_row] <- 1

> # if it was the expected ROI

> if (priorID==1) {

> face_fixations_morphs$ROI_type[i_row] <- 1

> # if it was the unexpected ROI

> } else if (unexpectedID==1) {

> face_fixations_morphs$ROI_type[i_row] <- 2

> # if it was one of the other two ROIs

> } else if (unexpectedID!=1) {

> face_fixations_morphs$ROI_type[i_row] <- 3

> }

> }

> # dwell time

> # loop over participants, blocks, and trials

> # fixations on e.g. ROI#1

> face_fixations_morphs_ROI_1 <-

> face_fixations_morphs[(face_fixations_morphs$ROI==1) ,]

> # if there were fixations on ROI#1

> if (nrow(face_fixations_morphs_ROI_1)>0) {

> # dwell time on ROI#1

> face_fixations_morphs_ROI_1$dwell_time <-

> sum(face_fixations_morphs_ROI_1$duration)

> } else {

> # if there were no fixations on ROI#1

> face_fixations_morphs_ROI_1$dwell_time <- 0

> }

## Expected outcomes

After performing all analyses described above, the expected outcomes are 1) count data of saccades per ROI and condition (expected, unexpected), 2) ordinal numbers of fixations on all ROIs, and 3) number of fixations and dwell time per ROI and condition (expected, unexpected, other two).

## Quantification and statistical analysis

1. Test the influence of priors on initial visual sampling.
  a. To test whether initial visual sampling is influenced by priors, define how many of the initial saccades you want to consider for the analysis.

***Note:*** In our experiment, we tested whether significantly more first or second saccades^11^ are performed towards the ROI with the expected facial feature (hereafter: ‘expected ROI’) compared to the same ROI if its feature was unexpected by conducting a 2 ✕ 2 repeated measures ANOVA with the within-subject factors ‘expectation’ (expected, unexpected) and ‘saccade’ (first, second).

***Note:*** You can use averaged relative frequencies to account for the different number of trials in which the facial feature of an ROI (e.g., the nose) was expected vs. unexpected (e.g., 1:3), yielding mean percentages for each participant for the different combinations of ‘saccade’ and ‘expectation’.

***Note:*** You could simply investigate the first saccade.^11^ Investigating the first two saccades allowed us to identify interactions with expectations, e.g., the first saccade landed more often in an ROI if its facial feature had been expected compared to the second saccade in trials in which it had not been expected.

2. Evaluate the order effect.
  ***Note:*** There are two approaches to evaluating an order effect.
  a. Firstly, in all trials (i.e., *match*, *mismatch*, *partial*), test whether participants fixated on the expected ROI earliest out of all the four ROIs.
    i. In case of missing fixations, assign ROIs that were not fixated randomly to one of the missing ordinal numbers in that trial.^12^
    ii. Evaluate whether the distribution of ordinal numbers differs from a uniform distribution using a subject-level chi-square goodness of fit test (R-package ‘htestClust’^13^) and calculate Cramer’s *V* as an effect size.
    iii. Perform post-hoc proportional tests for clustered data, corrected for the number of tests.
    iv. As an estimate of effect size, average the subject-level Cohen’s *h.*
  b. Secondly, in *partial* and *mismatch* trials, separately, test whether the expected ROI, the ROI with the unexpected facial feature (i.e., ‘unexpected ROI’), or the other two ROIs (i.e., ‘other ROIs’) are fixated more often before the other.
    i. This can be done by calculating tests of marginal proportion for clustered data against binomial distributions,^13^ tested against the random proportion of fixations depending on the number of ROIs, respectively, to account for the area of the other ROIs.
      ***Note:*** This analysis involves the assignment of random numbers in case of missing fixations. To ensure reproducible results, set the seed for each participant to a reproducible number.
3. Evaluate the prior effect on the number of fixations.
  a. Evaluate an expectation effect on the number and duration of fixations by using paired *t*- or Wilcoxon signed-rank tests (two-tailed), when the requirements for a *t*-test are violated.
  b. Test whether the number of fixations and/or the dwell time on the expected ROI differs from the unexpected ROI and the average across the other ROIs.
  c. Divide the number of fixations and dwell times on each ROI by the total number of fixations and face presentation duration, respectively, to yield proportions.

## Limitations

The results acquired using this analysis strategy can be impaired by different factors. These include noisy data, problems during seed ROI definition, and insufficient statistical power.

The order of fixations may be additionally influenced by unintended viewing strategies. For example, in our data, the first fixations landed predominantly in the center of the face, in line with a central viewing tendency.^14^^,^^15^ The central viewing tendency may reflect a general prior expectation of detecting important information in the center of an image. Indeed, it is reasonable that humans use this general prior expectation to place their first fixation. However, this tendency may also reflect an orienting response that allows the positioning of the next saccade/fixation at a location that is expected to be informative. Therefore, this central tendency does not limit the usability of our cue-based experimental approach.

Also, note that hardware issues, such as difficulties in measuring participants with glasses or heavy eye makeup, may necessitate the exclusion of certain individuals from these experiments for the foreseeable future.

## Troubleshooting

### Problem 1

Sometimes the calibration of the eye tracker does not work properly (e.g., due to dense eyelashes).

### Potential solution

We intended to use a 13-point-calibration grid, if possible. A calibration with fewer points might lead to decreased accuracy of measured eye movements. However, when the 13-point-calibration failed, we reduced the number of calibration points to a 9-point- or even a 5-point-calibration. Additional potential solutions for poor calibration can be achieved by adjusting the pupil and corneal reflection thresholds or the camera angle.

### Problem 2

Eye movements may only be attracted by salient visual features in an image, i.e., bottom-up information, so that priors do not show an effect.

### Potential solution

Choose visual stimuli carefully and account for stimulus properties such as luminance, size, etc. Consider that eye movements are guided by both the features of the visual stimulus as well as context or prior information. Salient stimulus features, such as color, intensity, and orientation contrast, attract eye movements^16^ and may, therefore, counteract prior-induced eye movements. Depending on the research question, balance the trade-off between controlling stimulus properties versus stimulus naturalness, as unnatural images may also be useful in canceling out certain stimulus properties. In our experiment, we chose artificially generated face images that did not contain additional salient features such as emotional expressions, scars, or any accessories such as glasses or facial piercings.

### Problem 3

Unsuccessful training so that participants do not learn the cue-stimulus associations.

### Potential solution

To measure prior effects on eye movements, make sure that participants actually learned the cue-stimulus association. Pre-specify your exclusion criterion accordingly. During data collection, we noticed that a few participants did not sufficiently learn the four identities and their distinct features in the training. We defined a cut-off threshold of 75% and allowed participants to repeat the last part of the training up to two times.

### Problem 4

When you extract fixations from the logfile, there are sometimes reading-in errors in the eyelinkReader toolbox. The toolbox extracts information about fixations, saccades, and blinks from the events ‘ENDFIX’, ‘ENDSACC’, and ‘ENDBLINK’. For example, we encountered duplicates of ENDSACC-events, while the second ENDSACC contained incorrect (implausibly high) time stamps for the relative onset time (‘sttime_rel’).

### Potential solution

We recommend performing sanity checks by checking the number of START and END events for fixations, saccades, and blinks. An imbalance might indicate incorrect duplicates. You can also manually check the entries in the read-in eye-tracking data. We solved this problem by looping through the fixations, saccades, and blinks and only considering data from END events that have preceding START events.

### Problem 5

The EyeLink 1000 manual mentions that blinks can be surrounded by preceding and following partial pupil occlusion that causes ostensible changes in pupil position. These are detected as saccades surrounding blinks and should be discarded as invalid data.

### Potential solution

To ensure stable pupil detection, time periods shortly before and after a blink might need to be discarded or interpolated. It could be helpful to consider the pupil diameter trace or the ratio of vertical to horizontal diameter to identify blink-associated artifacts.

## Resource availability

### Lead contact

Further information and requests for resources and reagents should be directed to and will be fulfilled by the lead contact, Helen Blank (helen.blank@rub.de).

### Technical contact

Questions about the technical specifics of performing the protocol should be directed to and will be fulfilled by the technical contact, Helen Blank (helen.blank@rub.de).

### Materials availability

All study materials have been deposited at the OSF and are publicly available (Experiment 1: https://osf.io/7e38v/; Experiment 2: https://osf.io/tbdh6/).

### Data and code availability

• Behavioral and eye-tracking data have been deposited at the OSF and are publicly available (Experiment 1: https://osf.io/7e38v/; Experiment 2: https://osf.io/tbdh6/).
• All original code has been deposited at the OSF and is publicly available (Experiment 1: https://osf.io/7e38v/; Experiment 2: https://osf.io/tbdh6/).

## Acknowledgments

This project was funded by the 10.13039/501100001659Deutsche Forschungsgemeinschaft (DFG, German Research Foundation) through the Emmy Noether program (grant no. DFG BL 1736/1-1 to H.B.).

## Author contributions

H.B.: writing – original protocol draft and funding acquisition; A.G.: writing – review and editing; M.L.: writing – review and editing; M.G.: writing – review and editing.

## Declaration of interests

The authors declare no competing interests.
